# The Burden of Mental Disorders in the Eastern Mediterranean Region, 1990-2013

**DOI:** 10.1371/journal.pone.0169575

**Published:** 2017-01-17

**Authors:** Raghid Charara, Mohammad Forouzanfar, Mohsen Naghavi, Maziar Moradi-Lakeh, Ashkan Afshin, Theo Vos, Farah Daoud, Haidong Wang, Charbel El Bcheraoui, Ibrahim Khalil, Randah R. Hamadeh, Ardeshir Khosravi, Vafa Rahimi-Movaghar, Yousef Khader, Nawal Al-Hamad, Carla Makhlouf Obermeyer, Anwar Rafay, Rana Asghar, Saleem M. Rana, Amira Shaheen, Niveen M. E. Abu-Rmeileh, Abdullatif Husseini, Laith J. Abu-Raddad, Tawfik Khoja, Zulfa A. Al Rayess, Fadia S. AlBuhairan, Mohamed Hsairi, Mahmoud A. Alomari, Raghib Ali, Gholamreza Roshandel, Abdullah Sulieman Terkawi, Samer Hamidi, Amany H. Refaat, Ronny Westerman, Aliasghar Ahmad Kiadaliri, Ali S. Akanda, Syed Danish Ali, Umar Bacha, Alaa Badawi, Shahrzad Bazargan-Hejazi, Imad A. D. Faghmous, Seyed-Mohammad Fereshtehnejad, Florian Fischer, Jost B. Jonas, Barthelemy Kuate Defo, Alem Mehari, Saad B. Omer, Farshad Pourmalek, Olalekan A. Uthman, Ali A. Mokdad, Fadi T. Maalouf, Foad Abd-Allah, Nadia Akseer, Dinesh Arya, Rohan Borschmann, Alexandra Brazinova, Traolach S. Brugha, Ferrán Catalá-López, Louisa Degenhardt, Alize Ferrari, Josep Maria Haro, Masako Horino, John C. Hornberger, Hsiang Huang, Christian Kieling, Daniel Kim, Yunjin Kim, Ann Kristin Knudsen, Philip B. Mitchell, George Patton, Rajesh Sagar, Maheswar Satpathy, Kim Savuon, Soraya Seedat, Ivy Shiue, Jens Christoffer Skogen, Dan J. Stein, Karen M. Tabb, Harvey A. Whiteford, Paul Yip, Naohiro Yonemoto, Christopher J. L. Murray, Ali H. Mokdad

**Affiliations:** 1 Institute for Health Metrics and Evaluation, Seattle, Washington, United States of America; 2 Preventive Medicine and Public Health Research Center, Department of Community Medicine, Iran University of Medical Sciences, Tehran, Iran; 3 Arabian Gulf University, Manama, Bahrain; 4 Iranian Ministry of Health and Medical Education, Tehran, Iran; 5 Sina Trauma and Surgery Research Center, Tehran University of Medical Sciences, Tehran, Iran; 6 Jordan University of Science and Technology, Irbid, Jordan; 7 Public Authority for Food and Nutrition, Kuwait, Kuwait; 8 Center for Research on Population and Health, Faculty of Health Sciences, American University of Beirut, Beirut, Lebanon; 9 Contech International Health Consultants, Lahore, Pakistan; 10 South Asian Public Health Forum, Islamabad, Pakistan; 11 Department of Public Health, An-Najah University, Nablus, Palestine; 12 Institute of Community and Public Health, Birzeit University, Ramallah, Palestine; 13 Harvard University, Boston, MA, United States of America; 14 Infectious Disease Epidemiology Group, Weill Cornell Medical College in Qatar, Doha, Qatar; 15 Health Ministers’ Council for Cooperation Council States, Riyadh, Saudi Arabia; 16 The Saudi Center for Evidence Based Healthcare, Riyadh, Saudi Arabia; 17 King Abdullah Specialized Children’s Hospital, King Saud bin Abdulaziz University for Health Sciences, Riyadh, Saudi Arabia; 18 Ministry of Health—Tunisia (Faculty of Medicine Tunis), Tunis, Tunisia; 19 Division of Physical Therapy, Department of Rehabilitation Sciences, Jordan University of Science and Technology, Irbid, Jordan; 20 University of Oxford, Oxford, United Kingdom; 21 Golestan Research Center of Gastroenterology and Hepatology, Golestan University of Medical Sciences, Gorgan, Iran; 22 Department of Anesthesiology, University of Virginia, Charlottesville, VA, United States of America; 23 Department of Anesthesiology, King Fahad medical city, Riyadh, Saudi Arabia; 24 OUTCOMES RESEARCH Consortium, Cleveland, OH, United States of America; 25 Hamdan Bin Mohammed Smart University, Dubai, United Arab Emirates; 26 Suez Canal University, Ismailia, Egypt; 27 Walden University, Minneapolis, MN, United States of America; 28 Federal Institute for Population Research, Wiesbaden, Germany; 29 Clinical Epidemiology Unit, Department of Clinical Sciences Lund, Orthopedics, Lund University, Lund, Sweden; 30 University of Rhode Island, Kingston, RI, United States of America; 31 SIR Consultants, Sindh, Pakistan; 32 School of Health Sciences, University of Management and Technology, Lahore, Pakistan; 33 Public Health Agency of Canada, Toronto, ON, Canada; 34 Department of Psychiatry, Charles R. Drew University of Medicine and Science, Los Angeles, California, United States of America; 35 David Geffen School of Medicine at UCLA, Los Angeles, California, United States of America; 36 Tehran University of Medical Sciences, Tehran, Iran; 37 London School of Hygiene & Tropical Medicine, London, United Kingdom; 38 Department of Neurobiology, Care Sciences and Society, Karolinska Institute, Stockholm, Sweden; 39 Bielefeld University, Bielefeld, Germany; 40 Department of Ophthalmology, Medical Faculty Mannheim, University Heidelberg, Heidelberg, Germany; 41 Department of Social and Preventive Medicine, School of Public Health of the University of Montreal, Montreal, Quebec, Canada; 42 Howard University College of Medicine, Howard University, Washington DC, United States of America; 43 Emory University, Atlanta, GA, United States of America; 44 University of British Columbia, Vancouver, BC, Canada; 45 Warwick—Centre for Applied Health Research and Delivery (WCAHRD), Division of Health Sciences, Warwick Medical School, University of Warwick, Coventry, United Kingdom; 46 Department of Surgery, University of Texas Southwestern, Dallas, Texas, United States of America; 47 Department of Psychiatry, American University of Beirut, Beirut, Lebanon; 48 Department of Neurology, Cairo University, Cairo, Egypt; 49 The Hospital for Sick Children, Toronto, Canada; University of Toronto, Toronto, Ontario, Canada; 50 Northern Territory Department of Health, Darwin, Northern Territory, Australia; 51 Centre for Adolescent Health, Murdoch Children’s Research Institute, Melbourne, Victoria, Australia; 52 Trnava University, Trnava, Slovakia; 53 University of Leicester, Leicester, United Kingdom; 54 Department of Medicine, University of Valencia/INCLIVA Health Research Institute and CIBERSAM, Valencia, Spain; 55 National Drug and Alcohol Research Centre, University of New South Wales, Sydney, New South Wales, Australia; 56 School of Public Health, University of Queensland, Herston, Queensland, Australia; 57 Queensland Centre for Mental Health Research, Wacol, Queensland, Australia; 58 Parc Sanitari Sant Joan de Déu—CIBERSAM, Sant Boi de Llobregat, Spain; 59 Nevada Division of Behavior and Public Health, Carson City, NV, United States of America; 60 National Institutes of Health, Bethesda, MD, United States of America; 61 Cedar Associates, Menlo Park, CA, United States of America; 62 Cambridge Health Alliance, Cambridge, MA, United States of America; 63 Federal University of Rio Grande do Sul, Porto Alegre, Brazil; 64 Department of Health Sciences, Northeastern University, Boston, Massachusetts, United States of America; 65 Southern University College, Johor, Malaysia; 66 Centre for Disease Burden, Norwegian Institute of Public Health, Bergen, Norway; 67 School of Psychiatry, University of New South Wales, Sydney, New South Wales, Australia; 68 Murdoch Children's Research Institute, Department of Paediatrics, University of Melbourne, Melbourne, Victoria, Australia; 69 Department of Psychiatry, All India Institute of Medical Sciences, New Delhi, India; 70 UGC Centre for Advanced Studies in Psychology, Utkal University, Vani Vihar, Bhubaneswar, Odisha, INDIA; 71 Department of Hospital Services, Ministry of Health, Phnom Penh, Cambodia; 72 Mental Health Association of Cambodia, Phnom Penh, Cambodia; 73 Department of Psychiatry, Stellenbosch University, Stellenbosch, South Africa; 74 Health and Life Sciences, Northumbria University, Newcastle upon Tyne, United Kingdom; 75 Domain for Mental and Physical Health, Norwegian Institute of Public Health, Bergen, Norway; 76 Center for Alcohol & Drug Research, Stavanger University Hospital, Stavanger, Norway; 77 Department of Psychiatry, University of Cape Town, Cape Town, South Africa; 78 MRC Unit on Risk & Resilience in Mental Disorders, Cape Town, South Africa; 79 School of Social Work, University of Illinois at Urbana-Champaign, Champaign, Illinois, United States of America; 80 University of Queensland, Brisbane, QLD, Australia; 81 Centre for Suicide Research and Prevention, The University of Hong Kong, Pok Fu Lam, Hong Kong; 82 Social Work and Social Administration Department, The University of Hong Kong, Pok Fu Lam, Hong Kong; 83 Department of Biostatistics, School of Public Health, Kyoto University, Kyoto, Japan; George Institute for Global Health, INDIA

## Abstract

The Eastern Mediterranean Region (EMR) is witnessing an increase in chronic disorders, including mental illness. With ongoing unrest, this is expected to rise. This is the first study to quantify the burden of mental disorders in the EMR. We used data from the Global Burden of Disease study (GBD) 2013. DALYs (disability-adjusted life years) allow assessment of both premature mortality (years of life lost–YLLs) and nonfatal outcomes (years lived with disability–YLDs). DALYs are computed by adding YLLs and YLDs for each age-sex-country group. In 2013, mental disorders contributed to 5.6% of the total disease burden in the EMR (1894 DALYS/100,000 population): 2519 DALYS/100,000 (2590/100,000 males, 2426/100,000 females) in high-income countries, 1884 DALYS/100,000 (1618/100,000 males, 2157/100,000 females) in middle-income countries, 1607 DALYS/100,000 (1500/100,000 males, 1717/100,000 females) in low-income countries. Females had a greater proportion of burden due to mental disorders than did males of equivalent ages, except for those under 15 years of age. The highest proportion of DALYs occurred in the 25–49 age group, with a peak in the 35–39 years age group (5344 DALYs/100,000). The burden of mental disorders in EMR increased from 1726 DALYs/100,000 in 1990 to 1912 DALYs/100,000 in 2013 (10.8% increase). Within the mental disorders group in EMR, depressive disorders accounted for most DALYs, followed by anxiety disorders. Among EMR countries, Palestine had the largest burden of mental disorders. Nearly all EMR countries had a higher mental disorder burden compared to the global level. Our findings call for EMR ministries of health to increase provision of mental health services and to address the stigma of mental illness. Moreover, our results showing the accelerating burden of mental health are alarming as the region is seeing an increased level of instability. Indeed, mental health problems, if not properly addressed, will lead to an increased burden of diseases in the region.

## Introduction

Mental disorders are a major public health concern. The global lifetime prevalence of mental disorders in adults is estimated to be between 12·2%-48·6% and the 12-month prevalence between 4·3% and 26·4% [1, 2]. According to the 2001 World Health Report, 10–20% of children and adolescents globally suffer from a mental disorder and 50% of mental disorders start before 14 years of age [3]. Mental disorders are also a leading contributor to the global disease burden: in 2013, 5·4% of global DALYs (Disability-Adjusted Life Years) and 17·4% of global YLDs (Years Lived with Disability) were due to mental disorders [4]. About 90% of suicides are due to underlying mental illness [5] with the World Health Organization (WHO) estimating an incidence of 800,000 suicides per year in 2005, with 86% being in low and middle-income countries [6]. Mental and substance use disorders were responsible for 22·5 million of the 36·2 million DALYs allocated to suicide in 2010 with depression contributing the most (46·1%) to suicide DALYS [7].

The Eastern Mediterranean Region (EMR) is a WHO-defined group of countries comprising Afghanistan, Arab Republic of Egypt, Bahrain, Djibouti, Iraq, Islamic Republic of Iran, Jordan, Kingdom of Saudi Arabia, Kuwait, Lebanon, Libya, Morocco, Oman, Pakistan, Palestine, Qatar, Republic of Yemen, Somalia, Sudan, Syrian Arab Republic, Tunisia, and the United Arab Emirates [8, 9]. The EMR has a population of about 583 million people [8]. Countries in the EMR vary significantly in terms of their gross domestic product, sociodemographic profiles, health indicators, and health system capacities and coverage [9].

Mental disorders are common in the EMR [10, 11]. Large scale community surveys conducted in the region report rates of psychological distress between 15·6% and 35·5%, with higher rates in countries with complex emergency situations. The 12-month prevalence of mental disorders ranges between 11·0% and 40·1% [12–23]. Depression and anxiety disorders are the most frequent mental disorders, and rates in women are up to double those in men [10].

Over the past two decades, the EMR has undergone significant improvements in health status, including increased life expectancy and reductions in child mortality [11, 24–26]. As people in the region are living longer, the burden of chronic diseases, including mental disorders, is expected to rise. Moreover, the more recent occurrences of conflict and violence in the region may further increase the prevalence and burden of mental disorders. Revolutions and change in government have occurred in Tunisia, Egypt, Yemen, and Libya. A war is currently devastating Syria with thousands of civilians forced to flee and seek refuge in camps in southern Turkey, Lebanon, and northern Jordan. Countless more have been displaced internally as well as internationally with the recent wave of both legal and illegal immigration to other parts of Europe [27, 28]. Political unrest is also common in Palestine, Iraq, Somalia, and Afghanistan. The violent deaths and injuries, population displacement, damage to infrastructure, rising unemployment and erosion of welfare systems that result from such conflict and violence can create fertile grounds for the development or progression of mental illness [29].

No prior studies have been conducted to quantitatively describe the burden of mental disorders in the EMR in 2013 in light of recent political changes in the region. In this study, we assess the fatal, non-fatal and total burden of mental disorders in the EMR in 2013 with reference to changes in burden since 1990 using data and methods from the Global Burden of Diseases, Injuries, and Risk Factors Study 2013 (GBD 2013).

## Methods

GBD 2013 estimated burden for 306 diseases and injuries and 79 risk factors. The DALY was used as a measure of total burden. One DALY represented one healthy year of life lost to a given disease or injury and was estimated by summing the fatal burden (as Years of Life Lost–YLLs) and non-fatal burden (as YLDs) associated with the disease or injury [30].

In GBD 2013, YLLs were computed by multiplying numbers of deaths from each cause in each age group by the reference life expectancy at the average age of death for those who die in the age group following the standard GBD 2010 methods [31–33]. Mortality rates and causes of death for each country-age-sex-year group were estimated in accordance with some general principles: identification of all available data sources, evaluation of the quality and correction for known bias in each data source, consistent statistical estimation including uncertainty analysis, and cross-validation analysis to assess model performance. Detailed information on the YLL estimation process has been published elsewhere [31–33].

YLDs were calculated as the sum of prevalent cases due to a given cause multiplied by disability weights–the general public’s assessment of the severity of health loss associated to the cause [4]. To estimate prevalence by age, sex, year and country, systematic reviews of the literature were conducted to capture data on the epidemiology of each mental disorder. Reported disorder-specific prevalence estimates were meta-analyzed using DisMod MR 2.0, a Bayesian meta-regression tool specifically designed for GBD purposes. Disability weights were derived using of the GBD 2010 method of pairwise comparison using population surveys conducted in Bangladesh, Indonesia, Peru, Tanzania, Hungary, Italy, Sweden, Netherlands, and the United States and an open-access internet survey. A disability weight ranging between 0 (equivalent to perfect health) and 1 (equivalent to death) was generated for 235 health states which together reflected all causes of non-fatal burden in GBD 2013. Detailed information on the YLD estimation process has been published elsewhere [4]. DALYs are computed by adding YLLs and YLDs for each age-sex-year-country group [30].

In this paper, we present GBD 2013 results for mental disorders, excluding substance use disorders. The GBD 2013 mental disorder grouping comprised of anxiety disorders, autistic spectrum disorders (autism and Asperger’s Syndrome), conduct disorder, eating disorders (anorexia nervosa and bulimia nervosa), schizophrenia, attention-deficit/hyperactivity disorder, bipolar disorder, depressive disorders (major depressive disorder and dysthymia), and idiopathic intellectual disability (a residual category capturing intellectual disability not attributed to any of the other causes in the study). We defined mental disorders according to criteria proposed in the Diagnostic and Statistical Manual of Mental Disorders and the International Classification of Diseases [4].

In an attempt to properly track the health status in the EMR countries, we divided the region into three categories according to the Gross National Income (GNI) *per capita*. The first category represents the low-income countries (LICs) with an average GNI *per capita* of $523 [4, 30–34]. On the opposite end of the spectrum are some oil-rich, high-income countries (HICs) with an average GNI *per capita* of $39,688. The nations that lie in between are the middle-income countries (MICs) with an average GNI *per capita* of $3,251. The three groups were LICs: Afghanistan, Djibouti, Republic of Yemen (Yemen), and Somalia; MICs: Arab Republic of Egypt (Egypt), Iraq, Islamic Republic of Iran, Jordan, Lebanon, Libya, Morocco, Pakistan, Palestine, Sudan, Syrian Arab Republic (Syria), and Tunisia; and HICs: Bahrain, Kingdom of Saudi Arabia (KSA), Kuwait, Oman, Qatar, and the United Arab Emirates (UAE).

## Results

In 2013, mental disorders in the EMR contributed to 11·9 million DALYs (95% Uncertainty Interval (UI): 7·6 million– 17·2 million) equivalent to 1894 DALYS/100,000 population. In comparison, mental disorders contributed to 1834 DALYs/100,000 globally in 2013.

Mental disorders were the leading cause of all nonfatal burden of disease (YLDs) in the EMR. In 2013, they accounted for 11·9 million YLDs or 19·0% of nonfatal burden. Globally, mental disorders contributed to 17·4% of nonfatal burden in 2013, ranking as second-largest contributor to nonfatal burden following musculoskeletal disorders. Depressive disorders accounted for most YLDs followed by anxiety disorders, both in the EMR and globally.

**Fig 1:**
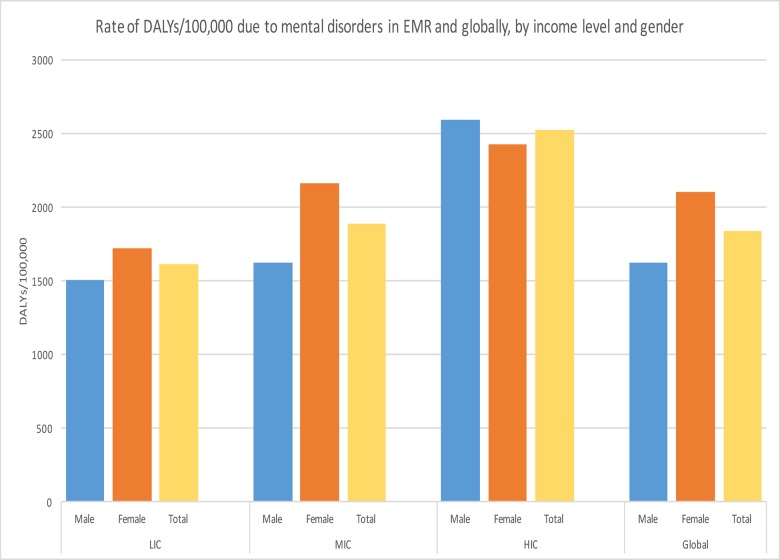
Rate of DALYs due to mental disorders in EMR and globally, by income level and gender.

In HICs of the EMR, mental disorders contributed to 2519 DALYs/100,000 of the population (2590/100,000 in males and 2426/100,000 in females). In MICs mental disorders contributed to 1884 DALYs/100,000 (1618/100,000 in males and 2157/100,000 in females). In LICs, mental disorders contributed to 1607 DALYs/100,000 (1500/100,000 in males and 1717/100,000 in females). Globally, mental disorders contributed to 1623 DALYs/100,000 in males and 2099 DALYs/100,000 in females (Fig 1).

**Fig 2:**
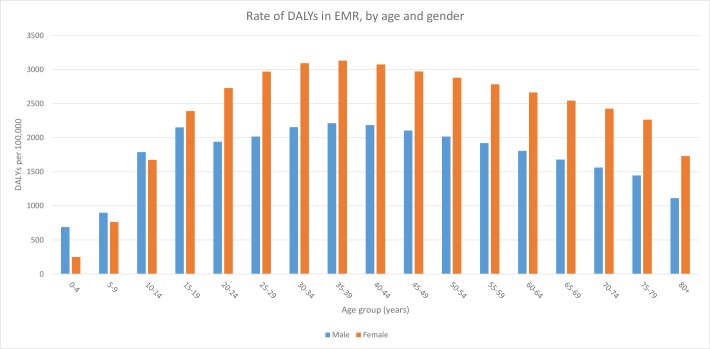
Rate of DALYs in EMR, by age and gender.

Females had a greater proportion of total burden attributable to mental disorders than males of equivalent ages, except for those under 15 years of age (Fig 2).

**Fig 3:**
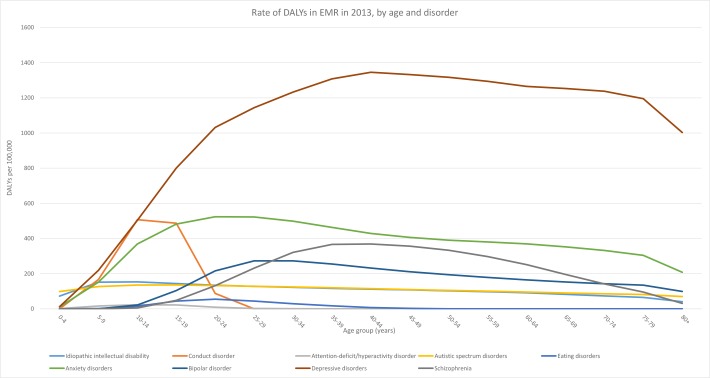
Rate of DALYs in EMR in 2013, by age and disorder.

The burden of mental disorders spanned all age groups. The highest rate of DALYs occurred in the 25–49 age group, with a peak in the 35–39 years age group for both sexes combined (5344 DALYs/100,000), females (3131 DALYs/100,000) and males (2213 DALYs/100,000) (Fig 1). The age pattern in males and females was different. In females, DALY rates increased progressively from birth up to age 35–39 (3131 DALYs/100,000) and then decreased incrementally with age. In males, DALY rates peaked at age 15–19 (2151 DALYs/100,000) and again at age 35–39 (2213 DALYs/100,000). The burden associated with depressive disorders and anxiety disorders rose abruptly in adolescence. Depressive disorder burden peaked between 40–44 years (1346 DALYs/100,000). Anxiety disorder burden peaked between 20–29 years (523 DALYs/100,000). Schizophrenia burden peaked between 35–44 years (367 DALYs/100,000) and bipolar disorder peaked between 25–34 years (273 DALYs/100,000). Conduct disorder peaked between 10–14 years (507 DALYs/100,000) and intellectual disability peaked between 5–24 years (range between 134–153 DALYs/100,000). ADHD occurred mainly between 5–19 years (range between 16–23 DALYs/100,000) (Fig 3).

**Fig 4:**
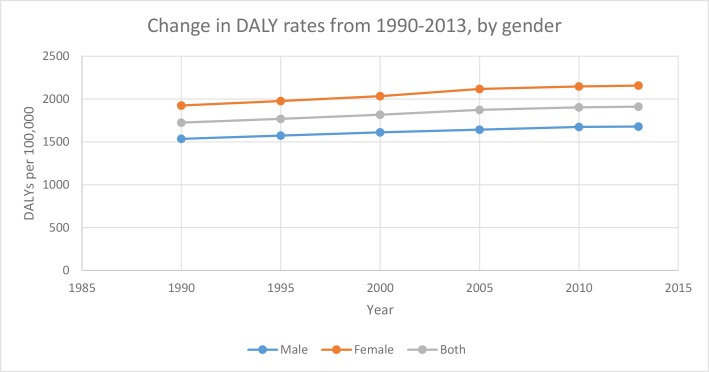
Change in DALY rates from 1990–2013, by gender.

**Fig 5:**
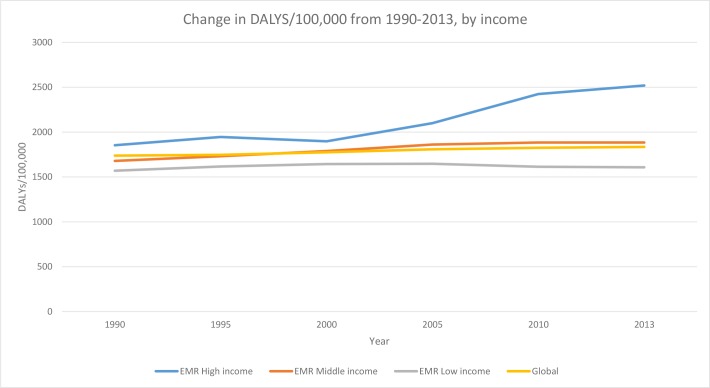
Change in DALYS/100,000 from 1990–2013, by income.

The burden of mental disorders in EMR increased from 1726 DALYs/100,000 in 1990 to 1894 DALYs/100,000 in 2013 (9·7% increase) (Fig 4). Globally, it increased from 1738 DALYs/100,000 to 1834 DALYs/100,000 (5·5% increase) (Fig 5).

Among income groups in the EMR, low-income countries have had a relatively stable burden of mental disorders from 1990–2013. The burden of mental disorders in middle-income EMR countries, much like the global burden of mental disorders, has seen a slight increase. High-income EMR countries increased from 1990–1995 followed by a decrease from 1995–2000, to be followed by a sharp increase in burden between 2000 and 2005 and then a slower rise between 2010–2013.

**Fig 6:**
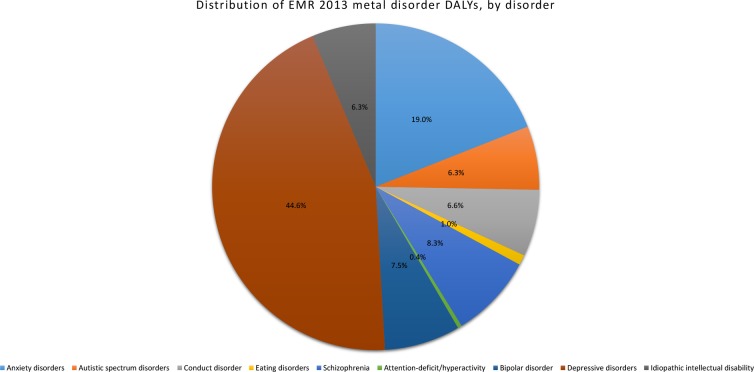
Proportion of DALYs explained by each mental disorder within the EMR.

Within the mental disorders group in EMR, depressive disorders accounted for most DALYs, followed by anxiety disorders (Fig 6) and then schizophrenia, bipolar disorder, conduct disorder, idiopathic intellectual disability, and autism spectrum disorders.

**Fig 7:**
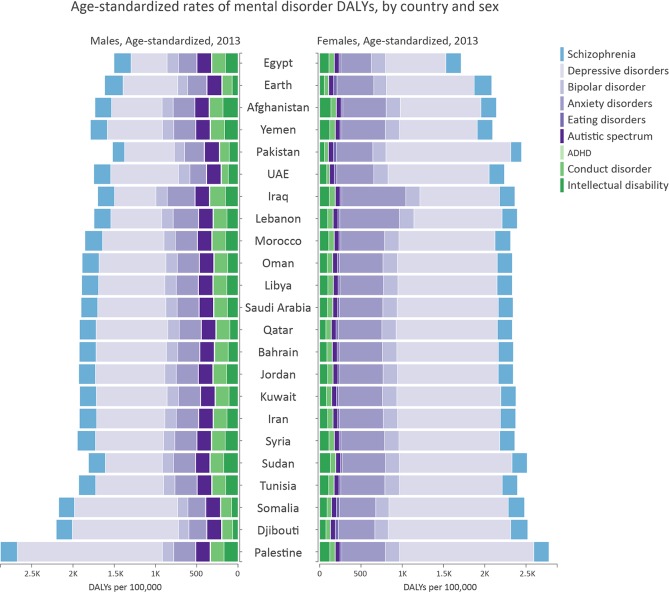
Age-standardized rates of mental disorder DALYs, by country and sex.

Among EMR countries, Palestine had the biggest burden of mental disorders. All EMR countries had a higher mental disorder burden compared to the global level, except for Egypt (Fig 7).

Mental disorders in the EMR contributed to 14,400 YLLs or 0·01% of total YLLs in EMR. YLLs are caused by schizophrenia and eating disorders. Mental disorder YLLs (as a proportion of total YLLs) were highest in the UAE, followed by Qatar. For eating disorders, YLLs were highest for UAE, Qatar, and Kuwait. For schizophrenia, YLLs were highest for Syria, Morocco, Iran, and Djibouti. Females had a higher percent of total YLLs due to mental disorders compared to males. The age group with the highest proportion of total YLLs due to mental disorders was the 25–29 age group followed by 30–34 age group.

## Discussion

This is the first study to comprehensively investigate the burden of mental disorders in the EMR. The burden of mental disorders was higher in almost all EMR countries compared to the global average.

EMR has seen a variety of progressive, overlapping challenges due ongoing wars and unrest. Historically, the region has been able to maintain a good health performance due to factors like a growing private health sector and resilience of the population [11, 25, 26, 35, 36]. Indeed, this burden is likely to increase due to the unrest [37]. Moreover, the countries in the region are not equipped to deal with this increase in burden. Hence, mental health will pose a major challenge and strain on resources in the coming years.

The literature is very rich in studies assessing the role that traumatic events in the EMR play in increasing risk of developing mental illness [23, 38–41]. Indeed, Palestine- which has seen almost 50 years of conflict, has the highest burden due to mental disorders in the EMR. 54·4% of Palestinian boys and 46·5% of Palestinian girls (age 6–12 years) were estimated to have emotional and behavioral disorders [42, 43]. However, the burden of mental disorders in Palestine should be interpreted with great caution, as it may be affected by a conflation between mental disorder and mental health, measurement validity issues [44], data accuracy issues [45], as well as surveys conducted post Israeli invasions of the Gaza Strip with symptoms declining over time. General and chronic exposure to trauma and violence related to the military occupation of Palestine, may also explain the high burden of mental disorders [46]. Moreover, 37·4% of Iraqi schoolchildren were estimated to be suffering from mental disorders and 22·2% of Afghani schoolchildren were estimated to have mental disorders [47–49]. Mental distress at such an early age further worsens the future projected burden. Syria, with its ongoing war, is also high on the burden spectrum, and there are grounds for being concerned that this will continue to rise. A recent study found a 33·5% prevalence of Post-Traumatic Stress Disorder among Syrian refugees in Turkey especially female refugees, who were exposed to two or more traumatic events and had a personal or family history of a psychiatric disorder [50]. However, Yemen, Iraq and Libya, which also have complex emergency situations as identified by WHO, are not high on the burden spectrum. This could be explained by the lack of quality data or any data coming from these countries. As mentioned earlier, these countries also have a deficiency in mental health professionals to diagnose and treat such conditions. Moreover, it is important to note that people in conflict zones living in unstable times tend to focus on staying alive and dealing with their daily needs of shelter and food. Such intense daily distraction could delay the manifestation of mental illness until the conflict is over.

Given the cultural specificities of the region, factors unrelated to war also contribute to mental disorders in EMR. For instance, polygamy is more common in Muslim communities, and women in polygamous marriages report a higher rate of depression and psychoticism than women in monogamous marriages [51]. This is in addition to the “first wife syndrome” seen in polygamous marriages, in which the first wife reports more anxiety, paranoid ideation, and psychoticism compared to the second and third wives [51]. Moreover, health and social inequalities for refugees across the EMR result in deprivation-related multi-morbidity including mental illness [52]. Intimate partner violence is also prevalent across much of the EMR [53].

Mental health services expenditure in the EMR was found to be insufficient by the WHO-Assessment Instrument for Mental health Systems (WHO-AIMS) study. The region’s per capita expenditure on mental health (US$ 0·15) is half that of global levels with only 2% of the government health budget allocated to mental health [8]. This level of spending is observed across low, middle and high income countries of the EMR. For instance, Qatar allocates 1% of total health expenditure on mental health services, Egypt allocates less than 1%, and Palestine allocates about 2·5%^60^[54]. Morocco devotes the highest percentage to mental health (about 4%), whereas Afghanistan, Pakistan, and Somalia allocate little to no money for mental health services [8]. This is compared to 6·3% in the US in 2009 [55]. Half of the mental health funds in the region go to mental hospitals, compared to a global proportion of 80% [56].

The provision of mental health services in the EMR continues to be suboptimal. The resources allocated to the screening and treatment of mental health services are not commensurate with the burden of mental disorders [54]. According to WHO’s 2014 Mental Health Atlas, EMR has 7·3 mental health workers and 4·2 hospital beds per 100,000 heads of population (compared to 43·5 mental health workers and 35 hospital beds per 100,000 heads of population in EURO) [57]. A study published in 2005 shows that Lebanon has the highest provision of psychiatric services with 1 psychiatrist per 45,000 heads of population [58]. A more recent paper in 2012 reports the highest proportions of psychiatrists in Bahrain (5 per 100,000), Qatar (3·4 per 100,000) and Kuwait (3·1 per 100,000) [54]. Countries like Iraq, Libya, Morocco, Sudan, Syria, and Yemen have fewer than 0.5 psychiatrists per 100,000 persons [54].

Among the mental disorders both globally and in EMR, depressive disorders appear to be the highest contributors to nonfatal burden. Low mood, loss of motivation, and anhedonia–features typical of major depressive disorder–impact one’s ability to function in the community. The prevalence of major depressive disorder in various EMR countries has been previously described [22, 59–62]. Moreover, the literature is rich in studies assessing the impact of wars and conflicts on depression [37, 63].

Burden due to premature mortality is not reflected in GBD YLL estimates. This is due to the way causes of deaths are assigned in the International Classification of Diseases (ICD) death coding system used by GBD. Yet, evidence shows that people with mental disorders experience a significant reduction in life expectancy, with risk of mortality increasing with disorder severity [64, 65].

Females contribute more to the burden of mental disorders across all age groups over 14 years in the EMR. Females in general are more likely to suffer from mental disorders compared to males [66]. Several studies have shed light on women’s increased risk of mental disorders in the Arab world and Middle East [67–69]. Conduct disorder and ADHD on the other hands are more common in males aged under 14 years in both the EMR and globally [70].

Our findings show that the burden of mental disorders is highest in the high-income EMR countries. This trend of increased mental illness with increased living standards has been described in developed countries [71]. Strong, positive linear associations of Gross National Income per capita with any mental illness and with serious mental illness were found along with a strong linear correlation between the prevalence of any mental illness and serious mental illness and income inequality [71]. Although higher income countries may have the overall highest burden of disease due to mental illness, the range in lower income countries is much greater. With the recent decline in oil prices, one would expect the burden of mental disorders in rich countries to increase in the near future secondary to economic turndown[72]. It has been shown in the United States that with poverty and low socioeconomic status, mental and functional health deteriorate[73–77]. In contrast to lower income countries in complex emergency situations, people in higher income countries lead more stable lives which allows them to attend to their mental health needs.

Regardless of income group or gender, there is a clear trend of increasing burden of mental illness over the years from 1990–2013. Previous GBD studies have attributed this increase to population growth and aging rather than an increase in prevalence. To date, there is insufficient evidence to show an increasing trend in the prevalence rate of mental disorders globally. The Arab spring wars are relatively recent and afflicted countries need time to recover to be able to conduct epidemiologic surveillance of mental health disorders in more recent years. Also, the stigma attached to mental illness may cause an underreporting of cases. Moreover, patients who develop mental disorders typically wait for a long period of time before seeking healthcare. However, there are a number of studies that have been recently done to evaluate the impact of the Arab spring wars on mental health [78].

Our study has a number of limitations. Most countries in the EMR lack quality epidemiological data to describe the national prevalence and burden of mental disorders and to provide quality representative data input for the GBD estimations. Raw prevalence data was available for 13 of the 22 EMR countries: Egypt, Iran, Iraq, Jordan, Lebanon, Morocco, Oman, Pakistan, Palestine, Sudan, Syria, UAE, and Yemen. A list of all datapoints used in this study is available on our Global Health Data Exchange web page. When data were of poor quality or unavailable, we relied on our ensemble modelling techniques to generate the estimates using other available variables and the information for neighboring countries or countries with a similar health profile in the region. We validated all these models by using out of sample techniques. This methodology and its validation are published in details elsewhere[4, 30–34, 79]. Moreover, we have a visualization on our web that shows the data sources and the effect of each step of our analyses on the estimates. Whilst this allowed us to include all EMR countries in our burden of disease analysis, it is important for countries in the region to facilitate the collection of high quality epidemiological data for mental disorders. Disability weights in GBD studies intentionally capture health loss while not attempting to capture welfare loss and hence will not reflect the economic and familial effects of mental disorders. In addition, given the subjective nature of the symptoms of mental illness, many individuals in cultures that express mental disorders differently from the ICD diagnostic criteria will not be captured by GBD.

A recent article published in the Lancet Psychiatry [80] showed that currently used approaches in GBD underestimate the burden of mental illness by more than a third. Possible causes include overlap between psychiatric and neurological disorders, the grouping of suicide and self-harm as a separate category, conflation of all chronic pain syndromes with musculoskeletal disorders, exclusion of personality disorders from disease burden calculations, and inadequate consideration of the contribution of severe mental illness to mortality from associated causes.

Our study clearly shows that mental health is a pressing priority in the EMR. Moving forward, countries of the EMR need a more substantial application of the mental health Gap Action Programme introduced by WHO to enhance political commitment and development of policy and legislative infrastructure pertaining to mental health [2]. While more widespread application of the WHO's mhGAP would play an important role in health system reforms to reduce the MH treatment gap, broader health system changes, and increased prioritization and resources, are necessary. It is worth noting that the Region has made significant progress on encouraging countries to adopt a locally-adapted version of the WHO MH Action Plan[81]. Ministries of Health need to promote studies on the prevalence and epidemiology of mental disorders on a national and subnational level. Moreover, there is an urgent need to integrate mental health treatment in the comprehensive medical management of patients. Cost-effective interventions are available, including for low and middle income countries [82]. Recent evidence shows that integrating depression care and medical care in primary care settings can improve outcomes for both mental and physical illnesses [83]. Governments should provide incentives to hospitals to expand their mental health facilities or create new ones in order to meet future demand. Moreover, medical schools should incentivize students to specialize in psychiatry training to ensure an adequate level of expertise in countries. Reorientation of mental health services from institutional- to community-based services, and routine collection of data on resources and burden of mental disorders, is also needed. Finally, awareness campaigns are crucial to encouraging those suffering in silence to seek medical care and speak up about their illness.
